# The molecular immune modulator adenosine deaminase-1 enhances HIV specific humoral and cellular responses to a native-like HIV envelope trimer DNA vaccine

**DOI:** 10.21203/rs.3.rs-4139764/v1

**Published:** 2024-04-22

**Authors:** Michele A. Kutzler, Gina Cusimano, David Joyner, Emily Konopka, Roshell Muir, Philip Barnette, Melanie Guderian, Iván del Moral-Sánchez, Ronald Derking, Tom Bijl, Jonne Snitselaar, Photis Rotsides, Kyra Woloszczuk, Matthew Bell, Gabriela Canziani, Irwin Chaiken, Ann Hessell, Yannic Bartsch, Rogier Sanders, Elias Haddad

**Affiliations:** Drexel University College of Medicine; Drexel University College of Medicine; Drexel University College of Medicine; Drexel University College of Medicine; Drexel University College of Medicine; Oregon National Primate Research Center; Twincore; Amsterdam UMC; University of Amsterdam; Amsterdam University Medical Center; University of Amsterdam; Drexel University College of Medicine; Drexel University College of Medicine; Drexel University College of Medicine; Drexel University College of Medicine; Drexel University College of Medicine; Oregon Health & Science University; Twincore; Amsterdam UMC, University of Amsterdam; Drexel University

## Abstract

There is currently no prophylactic vaccine available for human immunodeficiency virus (HIV). Research efforts have resulted in improved immunogens that mimic the native envelope (Env) glycoprotein structure. Recently, a novel triple tandem trimer (TTT) platform has been used to generate a plasmid encoding Env immunogen (pBG505-TTT) that expresses only as trimers, making it more suitable for nucleic acid vaccines. We have previously demonstrated that adenosine deaminase-1 (ADA-1) is critical to the T follicular helper (TFH) function and improves vaccine immune responses *in vivo*. In this study, we demonstrate that co-delivery of plasmid-encoded adenosine deaminase 1 (pADA) with pBG505-TTT enhances the magnitude, durability, isotype switching and functionality of HIV-specific antibodies in a dose-sparing manner. Co-delivery of the molecular immune modulator ADA-1 also enhances HIV-specific T cell polyfunctionality, activation, and degranulation as well as memory B cell responses. These data demonstrate that pADA enhances HIV-specific cellular and humoral immunity, making ADA-1 a promising immune modulator for HIV-targeting vaccines.

## Introduction

Human immunodeficiency virus (HIV) remains a prominent global health threat for which no prophylactic vaccine is available. Although there have been many efforts to develop an HIV vaccine, its development has proven to be challenging. Due to the high sequence diversity and multiple routes of transmission of HIV, it is critical for an effective HIV vaccine to induce both circulating and mucosal broadly neutralizing antibodies (bNAbs) capable of neutralizing most HIV circulating strains. Properties of the HIV envelope (Env) protein, used for viral entry pose, additional challenges. HIV Env protein is extremely metastable and heavily glycosylated, resulting in very little access to immune recognition of neutralizing epitopes and subsequent immune evasion [1, 2].

Vaccine attempts using monomeric gp120 subunits have failed to induce bNAbs and did not prevent infection [3–5], necessitating the development of improved envelope vaccine immunogens. Extensive research efforts have resulted in immunogens, including SOSIP trimers, that closely mimic the native Env glycoprotein structure and consistently induce autologous neutralizing responses [2, 6, 7]. Previous studies using protein BG505-SOSIP vaccination have focused on humoral characterization with little cellular characterization, particularly CD8^+^ T cell responses[8, 9]. Recently, a novel triple tandem trimer (TTT) platform has been used to generate a plasmid encoding Env immunogen (pBG505-TTT) that expresses BG505 trimers, making it more suitable for nucleic acid vaccines [10]. A recent study with another native-like Env immunogen MD39, showed that MD39-encoding DNA vaccination elicited enhanced Env-specific humoral and cellular responses compared to MD39 protein. These results provided evidence that a DNA vaccine platform could be advantageous for driving anti-HIV responses over a protein subunit vaccine [11].

We have previously demonstrated that a novel molecular immune modulator, adenosine deaminase-1 (ADA-1) enhances the magnitude and durability of both cellular and humoral vaccine induced immune responses *in vivo* [12–14]. This has been demonstrated across several vaccine antigens including SARS-CoV-2 [13, 14] and monomeric gp160 HIV envelope [12] DNA vaccines. ADA-1 is necessary for normal immune function as exemplified by the severe-combined immunodeficiencies (SCID) that results from ADA-1 loss-of-function mutations [15]. Pegylated ADA is used therapeutically as an enzyme replacement therapy for the treatment of ADA-SCID [16]. The enzymatic function of ADA-1 is to catalyze the non-reversable deamination of adenosine into inosine therefore regulating both intracellular and extracellular adenosine concentrations. Independent of its enzymatic function, ADA-1 has also been shown to promote T cell proliferation and differentiation [17–19]. Importantly, our lab has recently identified a novel function of ADA-1. We have demonstrated that ADA-1 prolongs TFH survival and induces TFH differentiation making it unique in boosting germinal center (GC) function resulting in increased quality of antigen specific adaptive immune responses to vaccines [13, 20].

TFH cells are necessary for germinal centers and are critical drivers of processes such as somatic hypermutation, affinity maturation and isotype switching in B cells. BNAbs against HIV exhibit high levels of somatic hypermutation and extensive affinity maturation [21]. Circulating TFH frequencies and function have been correlated with bNAbs [21, 22] and when administered passively, bNAbs against HIV have been shown to be protective against HIV acquisition in both humans and non-human primates (NHP) [23, 24]. In addition to antibody (Ab) neutralization, Fcγ receptor (FcγR)-mediated Ab function such as Ab dependent complement deposition (ADCD) has been shown to decrease HIV replication and may play a role in HIV protection [25–30]. ADCD has been found to be primarily driven by non-neutralizing antibodies (nNAbs) in the context of HIV and such nNAbs have been found to act together with CD8^+^ T cells to confer heterosubtypic immunity in the context of other viral infections [31, 32].

CD8^+^ T cell function during chronic viral infections has been found to be sustained by TFH cells producing IL-21 [33]. CD8^+^ T cells present in elite controllers of HIV infection have been found to establish immunological synapses with infected CD4^+^ T cells, leading to IFN-γ production, degranulation, and the elimination of the target cells [34]. Vaccine strategies designed to enhance CD8^+^ T cell frequency and effector function have also demonstrated protective HIV immunity in NHP [35]. These data demonstrate the importance of CD8^+^ T cell responses in an effective HIV vaccine and how vaccine strategies targeting TFH cells can drive both humoral responses and cellular responses that are critical for protective HIV immunity.

Since ADA-1 enhances TFH cell differentiation and function [20], and TFH cells are central to many aspects critical for an effective anti-HIV humoral and cellular response, we believe ADA-1 would serve as a promising immune modulator for HIV targeting vaccines. We therefore hypothesized that ADA-1 combined with the improved HIV envelope antigen pBG505-TTT would result in enhanced magnitude and quality of both humoral and cellular HIV specific responses.

To evaluate this hypothesis, mice were immunized in the current study with pBG505-TTT alone or in combination with plasmid encoded ADA-1 (pADA). To understand how ADA-1 functions as an immune modulator in relation to more classically used adjuvants such as Alum and MF59, another cohort of mice were co-immunized with pBG505-TTT with or without pADA, recombinant BG505 protein (rBG505) and either Alum or an MF59-like adjuvant AddaVax. ADA-1 was found to enhance HIV specific humoral responses as demonstrated by increased SOSIP-specific antibody magnitude, durability, isotype switching and functionality. pADA co-immunization also resulted in enhanced HIV specific cellular responses including increased T cell cytokine production, polyfunctionality, and activation as well as memory B cell responses. In comparison to other adjuvants, pADA was able to enhance Ab neutralization compared to alum while AddaVax induced robust Ab neutralization with no further enhancement with pADA. This study provides a proof of concept that ADA-1 can be used as an effective immune modulator for HIV nucleic acid-based vaccine strategies.

## Materials and Methods

### DNA plasmid preparation

The untagged BG505 SOSIP.v8.1 gene, used to produce Env protein for vaccination (rBG505), was derived from the BG505 *env* gene (Genbank ADM31403.1) by introducing a subset of the MD39 trimer-stabilizing mutations (519S, 568D, 570H and 585H, HxB2 numbering) [36] and a stop codon after residue 664 to the previously described BG505 SOSIP.v5.2 sequence [37]. His-tagged and avi-tagged BG505 SOSIP.v8.1 genes, used to produce proteins for immune responses characterization, included C-terminal sequences encoding GSGSGGSGHHHHHHHH and GSGLNDIFEAQKIEWHE peptides, respectively. The underlined GS residues were encoded by a BamHI restriction site for cloning purposes. The untagged BG505 triple tandem trimer (TTT) gene, encoded in the Env DNA immunogen (pBG505-TTT), was designed by concatenating three single-chain BG505 SOSIP.v8.4 protomers via 11-residue glycine-serine flexible linkers, as previously described [10]. This gene included a stop codon after position 664 of the third protomer. All BG505 constructs were generated by site-directed mutagenesis or by restriction-ligation of genes codon-optimized and synthesized by Genscript (Piscataway, USA) into a pPI4 plasmid. The BG505 TTT-encoding pPI4 plasmid used for vaccination was purified from heat-shock transformed DH5 competent cells (ThermoFisher Scientific, Cat: 18265017) using an NucleoBond Xtra Midi endotoxin-free (EF) kit (Macherey-Nagel), resuspended in EF water, and stored at −20°C until use.

A codon-optimized DNA plasmid encoding murine ADA-1 (pADA) (NCBI gene 11486) was subcloned into the pVax expression vector and produced commercially (Genscript, Piscataway, NJ). *In vitro* studies revealed the expression of the ADA proteins after transfection of cell lines with the vaccine construct as previously described [12, 38].

### Protein production

BG505 SOSIP.v8.1 proteins (untagged, his-tagged and avi-tagged) were expressed in HEK293F cells and purified by PG151 immunoaffinity chromatography, similar to previously described [39]. Briefly, HEK293F cells (Invitrogen, Cat:R79009) maintained in FreeStyle Expression Medium (Gibco) were transiently transfected with Env and furin expression plasmids in a 4:1 (w/w) Env to furin ratio. For transfection, the DNA mix was incubated with PEImax (Polysciences Europe GmBH, Eppelheim, Germany) in a 3:1 (w/w) PEImax to DNA ratio and then added to the supernatant of cells at a density of 0.8–1.2 million cells/mL. Five to seven days after transfection, supernatants were harvested, centrifuged and vacuum-filtered using 0.22 μm Steritops (Millipore, Amsterdam, The Netherlands). Proteins in the filtered supernatants were captured on PGT151-coupled CNBr-activated sepharose 4B beads (GE Healthcare) by flowing at 0.5–1.0 mL/min or overnight rolling incubation at 4°C. Subsequently, the beads were immobilized in Econo-Column chromatography columns (Biorad) and washed with three column volumes of a 0.5 M NaCl 20 mM Tris HCl pH 8.0 solution. After elution with 3 M MgCl2 pH 7.5, proteins were buffer exchanged to TN75 (75 mM NaCl, 20 mM Tris HCl pH 8.0) or PBS buffers using Vivaspin20 MWCO 100 kDa ultrafiltration units (Sartorius, Gӧttingen, Germany). After purification, avi-tagged proteins were biotinylated using a BirA500 biotin-ligase reaction kit (Avidity, Aurora, USA) according to manufacturer’s instructions. Excess biotin was removed by ultrafiltration with Vivaspin6 MWCO 10 kDa filters (Sartorius, Gӧttingen, Germany). Protein concentrations were determined using a NanoDrop One spectrophotometer (ThermoFisher Scientific) and molecular weight and extinction coefficient values calculated with the ProtParam webtool (Expasy). Purified proteins were filter sterilized and stored at −80°C until use. Untagged BG505 SOSIP.v8.1 protein was used for immunizations, his-tagged protein was used for ELISA assays, and avi-tagged biotinylated protein was used for memory B cell flow cytometry analysis.

### Immunizations

Female BALB/c mice aged 6–8 weeks were immunized in the tibialis anterior (TA) muscle with 20–40 μl of the formulated vaccines. Mice were immunized with 1 or 5 μg pBG505-TTT alone or were co-formulated with 10 μg pADA in the right TA muscle. Mice that were co-immunized with both pBG505-TTT and protein received 10 μg pBG505-TTT alone or with 10 μg pADA in the right TA muscle and 10 μg of recombinant BG505 protein (rBG505) co-formulated with protein adjuvants Alum (Aluminum hydroxide gel, Invivogen, Cat:vac-alu-250) or AddaVax (Invivogen, Cat:vac-adx-10) in the left TA muscle. Alum and MF59 were formulated at a 1:1 volume ratio with antigen (i.e. 65.5 μL rBG505 + 65.5 μL Alum or AddaVax), per the manufacturer’s recommendation. Mice that were immunized with protein alone received 10 μg rBG505 co-formulated with Alum or AddaVax in the right TA muscle (see **Supplemental Fig. 1c**). Control mice were immunized with 15 μg or 20 μg of empty plasmid vector (Pvax) to ensure an equal amount of DNA was administered across experimental groups. Immediately after DNA vaccine injection, *in vivo* electroporation was performed using the CELLECTRA device (Inovio Pharmaceuticals, Bluebell, PA). Animals were housed in a temperature-controlled, light-cycled, specific-pathogen-free facility at Drexel University College of Medicine in accordance with protocols approved by Drexel University Institutional Animal Care and Use Committee. Previous studies immunizing male and female mice with pADA did not show any sex biases nor any species (C57BL/6 or BALBc) biases [13].

### Mouse sacrifice, sample collection, and tissue harvest

**Figure 1:**
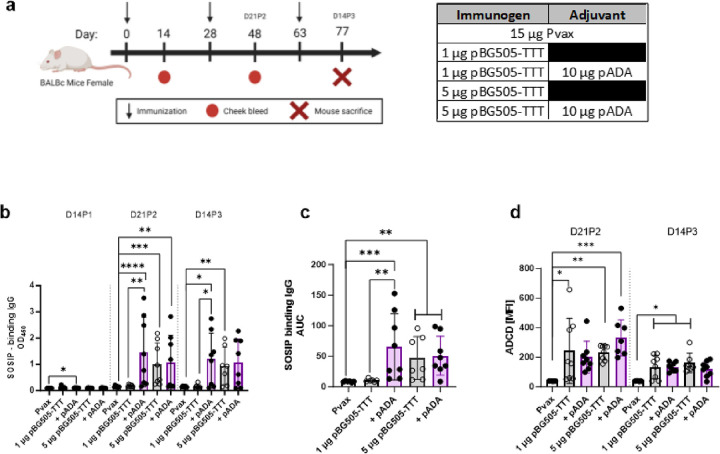
ADA-1 enhances HIV specific IgG and Antibody Dependent Complement Deposition (ADCD). Female BALB/c mice were immunized thrice with 1 μg or 5 μg of pBG505-TTT alone or with 10 μg of DNA-encoded ADA-1 (pADA). Control mice were immunized with 15 μg of the empty backbone Pvax. Mice were bled at D14P1 and D21P2 immunizations prior to sacrifice at D14P3 immunizations **(a)**. Sera was used to determine SOSIP specific IgG responses via ELISA (**b**). Area under the curve (AUC) was calculated for SOSIP specific IgG across all timepoints (D14P1, D21P2 and D14P3) **(c)**. Sera was used to determine antibody dependent complement deposition (ADCD) **(d).** Data are representative of one experiment. Symbols represent individual animals (n=8 per group). White bars with closed circles represent mice immunized with Pvax. Gray bars with open circles represent animals immunized with bars 1 μg or 5 μg of pBG505-TTT. Purple bars with closed circles represent animals that were immunized with 1 μg or 5 μg of pBG505-TTT + pADA. Bars represent the mean and error bars represent the standard deviations. *p<0.05, **p<0.01, ***p<0.001, and ****p<0.0001 by Mann-Whitney-U test **(b-c)** or Kruskal-Wallis ANOVA **(d)**.

At the time points shown in the *in vivo* study designs (Fig. 1a and Fig. 3a), mice were either bled via cheek bleed or sacrificed. At sacrifice, blood and spleens were collected. Blood collected via cheek bleed or cardiac puncture was collected into minicollect serum gel tubes (Grenier-Bio) and centrifuged at 16,000 rpm for 10 min at 4°C to separate serum. Separated serum was aliquoted and frozen at −80°C for subsequent use in ELISA, ADCD and neutralization assays. Spleens were processed into single-cell suspensions and resuspended in RPMI medium supplemented with 1% penicillin/streptomycin and 10% FBS. Cell concentrations and viabilities were determined using a Countess Automated Cell Counter (Invitrogen, Life Technologies).

### ELISA assays

ELISA was used to determine SOSIP-specific IgG, IgG1, IgG2a, IgG2b and IgG3 present in mouse serum. Mouse blood samples were collected via cheek bleed or cardiac puncture. Ni-NTA HisSorb plates (Qiagen, Cat:35061) were coated with 100 μl/well of recombinant his-tagged BG505-SOSIP.v8.1 protein diluted in 1xTBS (Sera Care or Fisher) to a concentration of 1–2 μg/ml and incubated at room temperature for 2 hrs. Plates were washed with 1xTBS before the addition of diluted mouse serum. For IgG and IgG1 ELISAs, mouse sera were diluted 1:100 in 1xTBS with 2% non-fat dry milk (Rockland Immunochemicals). For IgG2a, IgG2b and IgG3 ELISAs, mouse sera were diluted 1:25 in 1xTBS with 2% non-fat dry milk. Diluted serum was incubated at room temperature for 2 hrs. To ensure consistency from assay to assay, the human monoclonal Ab VRC01 (Cat: ARP-12033, NIH HIV reagent program) was added at a concentration of 500 ng/mL and serially diluted with 1xTBS with 2% non-fat dry milk to 7.8 ng/mL (see **Supplemental Fig. 1a-b**). Plates were washed with 1xTBS before incubation with HRP-conjugated secondary antibodies. HRP-conjugated goat anti-mouse secondary antibodies were diluted as follows in 1xTBS with 2% non-fat dry milk: IgG (Columbia Biosciences, Cat:HRP-112) 1:500, IgG1 (Invitrogen, Cat:A10551) 1:2000, IgG2a (Southern Biotech, Cat:1081–05) 1:4000, IgG2b (Southern Biotech, Cat:1091–05) 1:4000, and IgG3 (Southern Biotech, Cat:1101–05) 1:4000. HRP-conjugated goat-anti human IgG (Sigma, Cat:A0293–1ML) diluted 1:3000 in 1xTBS with 2% non-fat dry milk was added to VRC01 containing wells. HRP-conjugated secondary antibodies were added 100 μl/well and incubated at room temperature for 1hr. Plates were washed with 1xTBS with 0.05% Tween-20 and developed using TMB Ultra substrate (Thermo Fisher) according to the manufacturer’s instructions. SOSIP-specific antibody is represented as the average O.D. at 450 nm across duplicate wells.

### Area under the curve analysis

For each mouse, area under the curve (AUC) was determined for SOSIP-specific IgG across the following timepoints: day 14 post 1 immunization, day 21 post 2 immunizations, and day 14 post 3 immunizations. Outliers were determined and removed using the robust regression and outlier removal algorithm in GraphPad Prism. AUC was calculated using the AUC analysis function in GraphPad Prism. Statistical significances (p < 0.05) between different experimental group AUC values were determined by nonparametric Mann-Whitney U-test.

### Activation-induced marker assay

Splenocytes from vaccinated mice were cultured at 37°C with 5% CO_2_ for 18 hrs in the presence of a megapool of BG505 SOSIP-specific peptides at a concentration of 2.5μg/mL per peptide (Cat: ARP-13123, NIH HIV reagent program) in 96-well U-bottom plates at 1 × 10^6^ splenocytes per well. A stimulation condition with an equal percentage amount of DMSO was included as a negative control, while PMA/Ionomycin (eBioscience, Cat: 00-4978-93) was used as a positive control. Following stimulation, cells were stained with live/dead Fixable Aqua Dead Cell Kit (Cat: L34957; Invitrogen) and anti-mouse CD16/32 (Clone: 93; Cat:101319; BioLegend) for 20 mins at 4°C, protected from light. Cells were then surface stained for 45 mins at 4°C protected from light with the following fluorochrome-conjugated anti-mouse Abs: CD3 (Clone: 17A2, Cat: 100216), CD4 (Clone: GK1.5; Cat:100414), CD8 (Biolegend, Clone: 53 – 6.7, Cat: 100748), CD25 (Clone: PC61; Cat: 102010), OX40 (Clone: OX-86; Cat:119411), and PD-L1 (Clone: 10F.9G2; Cat: 124321) from BioLegend. Samples were acquired on a BD LSR Fortessa and data were analyzed using FlowJo software v.10 (BD Life Sciences).

### Memory SOSIP-specific B cell flow cytometry

Biotinylated BG505 protein was incubated on ice at a concentration of 0.2 μg/μL separately with either APC-streptavidin (Biolegend, Cat: 405207) or PE-streptavidin (Biolegend, Cat: 405204) at a concentration of 0.024 μg/μL for 1 hr shielded from light. Following 1 hr incubation, 100 μM of free biotin (Invitrogen, Cat:B20656) was added and allowed to incubate for 15 mins shielded from light at 4°C. Biotinylated BG505 protein incubated with PE-streptavidin was combined with biotinylated BG505 protein incubated with APC-streptavidin at a 1:1 ratio. Single-cell suspensions of splenocytes from vaccinated mice were added to 96-well V-bottom plates at 1 × 10^6^ splenocytes per well. Cells were stained for viability in the presence of Live/Dead Aqua (Invitrogen, Cat: L34957) and anti-CD16/CD32 (BioLegend, Clone: 93; Cat:101319) for 20 mins at 4°C. Cells were then washed and stained for 30mins at 4°C with 5 μL of the biotinylated BG505 protein probe and 95 μL of a surface antibody cocktail containing the following fluorochrome-conjugated anti-mouse Abs: CD3 (Biolegend, Clone: 17A2, Cat:100216), CD19 (Biolegend, Clone: 6D5; Cat: 115530), and IgD (Biolegend, Clone: 11–26c.2a; Cat: 405742). Samples were acquired on a BD LSR Fortessa and data were analyzed using FlowJo software v.10 (BD Life Sciences).

### T cell cytokine flow cytometry

Splenocytes from vaccinated mice were cultured at 37°C with 5% CO_2_ for 6 hrs in the presence of a megapool of BG505 SOSIP-specific peptides at a concentration of 2.5 μg/mL per peptide (NIH HIV reagent program, Cat: ARP-13123,) and fluorochrome-conjugated anti-CD107a (Biolegend, Clone:1D4B, Cat: 121606) in 96-well U-bottom plates at 1 × 10^6^ splenocytes per well. A stimulation with an equal percentage amount of DMSO was performed as a negative control, while PMA/Ionomycin (eBioscience, Cat: 00–4978-93) was included as a positive control. During stimulation, cells were also incubated with protein transport inhibitor (ebioscience, Cat: 00-4980-93). Following stimulation, cells were stained with Live/Dead Aqua Fixable Viability Stain (Invitrogen, Cat: L34957) and anti-mouse CD16/32 (BioLegend, Clone: 93; Cat:101319) for 20 mins at 4°C protected from light. Cells were then surface stained for 45 mins at 4°C protected from light with the following fluorochrome-conjugated anti-mouse Abs: CD3 (Biolegend, Clone: 17A2, Cat: 100216), CD4 (Biolegend, Clone: GK1.5; Cat:100414), CD8 (Biolegend, Clone: 53 – 6.7, Cat: 100748), CD62L (Biolegend, Clone:MEL-14, Cat:104448), and CD44 (Biolegend, Clone:IM7, Cat:103032). Following surface staining, cells were washed and resuspended in 100 μL BD CytoFix/CytoPerm^™^ solution (Cat:554722) for 1 hr at 4°C protected from light. Cells were intracellularly stained with the following fluorochrome-conjugated anti-mouse Abs diluted in 1x perm/wash buffer (BD, Cat: 554723) for 1hr at 4°C protected from light: IFNγ (Biolegend, Clone: XMG1.2; Cat: 505810), TNFα (Biolegend, Clone: MP6-XT22, Cat:506333) and IL-2 (BD, Clone: JES6–5H4; Cat: 503832). Samples were acquired on a BD LSR Fortessa and data were analyzed using FlowJo software v.10 (BD Life Sciences).

### ELISpot assay

PVDF membrane ELISpot plates (Mabtech, Cat: 3654WP10) were activated with 35% ethanol and washed with PBS prior to coating. Plates were coated with mouse anti-IFN-γ capture antibody (R&D, Cat: SEL485) and incubated overnight at 4°C. Plates were washed with PBS and blocked for 2 hrs at room temperature with PBS containing 1% BSA and 5% sucrose. Splenocytes from vaccinated mice were added (2×10^5^ cells/well) and stimulated with five BG505 SOSIP-specific peptide pools at a concentration of 2.5 μg/mL per peptide (Cat: ARP-13123, NIH HIV reagent program). A stimulation with an equal percentage amount of DMSO was performed as a negative control, while PMA/Ionomycin (ebioscience, Cat:00–4970-93) was included as a positive control. Cells were stimulated overnight at 37°C with 5% CO_2_. Plates were then washed and incubated with mouse IFN-γ detection antibody (R&D, Cat: SEL485) overnight at 4°C. Plates were washed and incubated with Streptavidin-ALP antibody for 2 hrs at room temperature prior to development. To develop, plates were incubated with BCIP/NBT substrate for 30 mins at room temperature protected from light. Plates were washed and left to dry overnight at room temperature. Plates were then scanned and counted using CTL ImmunoSpot S5 Core Analyzer (Cellular Technology Limited CTL).

### Neutralization assay

Pseudoviruses were produced using the pSG3ΔEnv DNA plasmid encoding the HIV backbone and a plasmid encoding (Q461e2TAIV) envelope as previously described [40]. Briefly, 293T cells were plated at 5 × 10^6^ cells/flask in a T-75cm flask in medium (DMEM, 10% fetal calf serum, 1% l-glutamine, 1% penicillin-streptomycin). The following day, 293T cells in DMEM without pen/strep were transfected with 10 μg total DNA/flask at a 20:1 ratio of backbone-to-Env plasmids in the presence of 20 μL polyethylenimine (jetPEI; Polyplus, Inc.) per flask. Cells were incubated for 48 hrs at 37°C. The virus was harvested by the centrifugation of the supernatant at 1,800 rpm for 10 min at 4°C. Pseudovirus stock was aliquoted and stored at −80°C until use. The pseudovirus was titrated on TZM-bl cells; dilutions of the pseudovirus were used to infect TZM-bl cells in the presence of 7.5 μg/ml DEAE-dextran (Fisher Biotech, Fair Lawn, NJ). Two days later, infection was measured by luciferase expression, and 50% tissue culture infective doses (TCID50) (determined by the Reed-Muench formula) were added to each neutralization assay.

The neutralization assays were performed in TZM-bl cells which have an HIV Trans-activator of Transcription (tat) induced luciferase reporter. A 3-fold dilution of the mouse serum was incubated with the tier 1A virus Q461e2TAIV for 1 hr at 37°C. TZM-bl cells were added with DEAE-Dextran and allowed to incubate for 48 hrs at 37°C with 5% CO_2_. Cells were lysed with Bright-Glo (Promega) for 2 mins before being read in a luminometer. ID_50_’s were calculated as a 50% reduction in the relative light units (RLU) in the mouse serum dilution wells as compared to the virus-only wells after subtraction of the cell-only controls. All samples were tested in duplicate, and response curves were fit by nonlinear regression.

### Antibody dependent cellular cytotoxicity (ADCD) assay

Antibody-dependent complement deposition (ADCD) was assessed as described previously [41]. Briefly, biotinylated BG505 protein was coupled to fluorescence NeutrAvidin beads (Thermo Fisher Scientific). Mouse sera antibodies were diluted 1:10 in 0.1% BSA and incubated with the coupled BG505 beads for 2 hrs at 37°C. Beads were washed and incubated with complement factors from guinea pig (Sigma-Aldrich) for 20 mins at 37°C. The complement reaction was then stopped by washing with 15 mM EDTA in PBS. C3 deposition on the beads was detected with a 1:100 diluted FITC-conjugated anti-guinea pig C3 polyclonal antibody (MP Biomedicals, Cat: 0855385) and relative C3 deposition was analyzed by flow cytometry.

### Statistical analysis

All statistics were analyzed using GraphPad Prism 9 or 10. Error bars represent means ± standard deviations. Outliers were determined and removed using the robust regression and outlier removal (ROUT) algorithm. Mann-Whitney U test or Kruskal-Wallis ANOVA statistical tests were performed where denotated to determine statistical differences between groups. In all data, *p < 0.05, **p < 0.01, ***p < 0.001, and ****p < 0.0001.

## Results

### ADA-1 enhances HIV specific IgG and Antibody Dependent Complement Deposition (ADCD)

To evaluate the effect of ADA-1 on HIV specific humoral responses, 6–8-week-old female BALB/c mice were immunized thrice with 1μg pBG505-TTT or 5 μg pBG505-TTT alone or in combination with 10 μg pADA. Control mice were immunized with 15 μg Pvax, an empty plasmid vector, to ensure mice received equal total amounts of DNA and to control for any nonspecific DNA-induced innate immune responses. Mice were bled on day 14 post 1 immunization (D14P1), day 21 post 2 immunizations (D21P2), and sacrificed on day 14 post 3 immunizations (D14P3) to evaluate SOSIP-specific antibody magnitude, durability, and function via ELISA, ADCD (Fig. 1a) or neutralization assays (**Supplemental Fig. 1d**). Consistency between ELISA assays was confirmed using the human monoclonal antibody VRC01 as a positive control for assay validation (**Supplemental Fig. 1a-b**).

Mice co-immunized with pADA and 1 μg pBG505-TTT exhibited significantly increased SOSIP-specific IgG (mean O.D.=1.482) compared to mice immunized with 1 μg pBG505-TTT alone (mean O.D.=0.184) at both D21P2 (**p = 0.0022) and D14P3 (*p = 0.0289). However, mice co-immunized with pADA and 5 μg pBG505-TTT exhibited comparable levels of SOSIP-specific IgG (mean O.D.=1.063) to mice immunized with 5 μg pBG505-TTT alone (mean O.D.=0.998) suggesting a plateau in the humoral response with 5 μg pBG505-TTT. The ability of pADA to significantly enhance humoral responses with 1 μg pBG505-TTT (mean O.D.=1.482) to levels comparable with 5 μg pBG505-TTT (mean O.D.=0.998) clearly demonstrates a dose sparing effect with pADA (Fig. 1b). To evaluate the durability of the SOSIP-specific IgG induced following immunization, an area under the curve (AUC) analysis was performed on SOSIP-specific IgG that was measured across all timepoints (D14P1, D21P2, and D14P3). Mice co-immunized with pADA and 1 μg pBG505-TTT (mean AUC = 65.47) exhibited significantly increased SOSIP-specific IgG over time (**p = 0.0027) compared to mice immunized with 1 μg pBG505-TTT alone (mean AUC = 10.36) suggesting that ADA-1 induces more durable humoral responses (Fig. 1c).

To evaluate the functionality of SOSIP-specific Ab, we performed an ADCD assay using sera from immunized mice at D21P2 and D14P3. At the D21P2 timepoint, mice co-immunized with pADA and 5 μg pBG505-TTT produced SOSIP-specific Ab with the most capacity to induce ADCD (mean MFI = 331.14) with a trend toward increased ADCD compared to mice that received 5 μg pBG505-TTT alone (mean MFI = 232.38), although not significant. Mice co-immunized with 1 μg pBG505-TTT and pADA (mean MFI = 201.48) did not exhibit increased ADCD compared to mice immunized with 1 μg pBG505-TTT alone (mean MFI = 243.39). Compared to pVax immunized mice, mice immunized with 1μg pBG505-TTT (*p = 0.0307), 5 μg pBG505-TTT (**p = 0.0038) and 5 μg pBG505-TTT with pADA (***p = 0.0001) had significantly increased ADCD. This suggests the ability of ADA-1 to modestly increase ADCD particularly when co-administered with 5 μg pBG505-TTT. At the D14P3 timepoint, ADCD capacity contracts across all immunization groups compared to the D21P2 timepoint and there is no difference between mice immunized with pBG505-TTT alone or co-immunized with pADA (Fig. 1d).

In addition to ADCD, Ab neutralization capacity was evaluated. Mice immunized 5 μg pBG505-TTT alone (***p = 0.0006, mean ID50 = 156.29) and with pADA (**p = 0.0014, mean ID50 = 127.59) had significantly increased neutralization against the tier 1A pseudovirus Q461e2TAIV compared to Pvax mice. However, there was no difference in neutralization capacity between mice that received pBG505 alone or with pADA (**Supplemental Fig. 1d**). Additionally, we observed no correlation between SOSIP-specific IgG magnitude and neutralization (**Supplemental Fig. 1e**). Although this study strictly used female BALBc mice, previous studies using pADA in male and female mice did not show any sex biases nor any species (C57BL/6 or BALBc) biases in humoral or cellular responses [13]. Together these data demonstrate that pADA is dose sparing, enhances SOSIP-specific IgG magnitude and durability as well as modestly increases the ability of SOSIP-specific antibody to induce ADCD.

### ADA-1 enhances HIV specific antibody isotype switching

**Figure 2:**
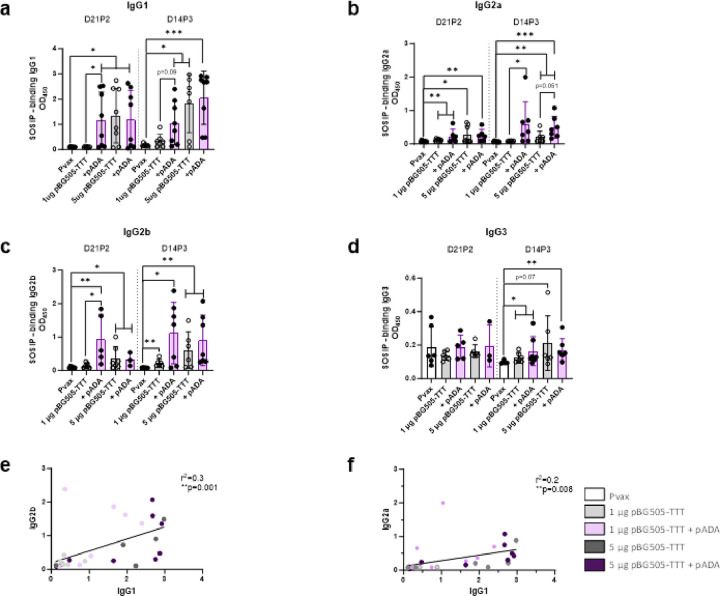
ADA-1 enhances HIV specific antibody isotype switching in a dose sparing manner. Mice were immunized and bled as outlined in Figure 1a. Sera was used to determine SOSIP specific IgG1 **(a)**, IgG2a **(b)**, IgG2b **(c)** and IgG3 **(d)** responses via ELISA. Pearson correlations were made between SOSIP specific IgG2a or IgG2b and IgG1 **(e-f)**. Data are representative of one experiment. Symbols represent individual animals (n=8 per group). White bars with closed circles represent mice immunized with Pvax. Gray bars with open circles represent animals immunized with bars 1 μg or 5 μg of pBG505-TTT. Purple bars with closed circles represent animals that were immunized with 1 μg or 5 μg of pBG505-TTT + pADA. Bars represent the mean and error bars represent the standard deviations. *p<0.05, **p<0.01, ***p<0.001, and ****p<0.0001 by Mann-Whitney-U test **(a-d).**

To evaluate if ADA-1 enhances isotype switching, we measured SOSIP-specific IgG1, IgG2a, IgG2b and IgG3 in the sera of immunized mice as shown in Fig. 1a. Mice co-immunized with pADA and 1 μg pBG505-TTT exhibited significantly increased SOSIP-specific IgG1 at D21P2 (*p = 0.0137, mean O.D.=1.186) and a near significant increase (p = 0.0939, mean O.D.=1.057) at D14P3 compared to mice immunized with 1 μg pBG505-TTT alone (mean O.D. D21P2 = 0.104, mean O.D. D14P3 = 0.348). Similar to total SOSIP-specific IgG, SOSIP-specific IgG1 levels plateau at 5 μg pBG505-TTT (mean O.D. D21P2 = 1.341, mean O.D. D14P3 = 1.821) and we observe no further enhancement with pADA (mean O.D. D21P2 = 1.208, mean O.D. D14P3 = 2.059) (Fig. 2a).

At D21P2 we observed little to no SOSIP-specific IgG2a in both pADA receiving (mean O.D. 1 μg pBG505-TTT + pADA = 0.230, mean O.D. 5 μg pBG505-TTT + pADA = 0.255) and non-receiving counterparts (mean O.D. 1 μg pBG505-TTT = 0.113, mean O.D. 5 μg pBG505-TTT = 0.269). However, at D14P3 we see that only mice that were co-immunized with pADA develop SOSIP-specific IgG2a (mean O.D. 1 μg pBG505-TTT + pADA = 0.594, mean O.D. 5 μg pBG505-TTT + pADA = 0.493). The increase in SOSIP-specific IgG2a in pADA co-immunized mice was significant when compared to mice receiving 1 μg pBG505-TTT alone (*p = 0.0192) and near significant when compared to mice receiving 5 μg pBG505-TTT alone (p = 0.0513). These data demonstrate the ability of ADA-1 to uniquely induce SOSIP-specific IgG2a which is not achieved with pBG505-TTT alone (Fig. 2b). When correlating SOSIP-specific IgG1 to IgG2a production, we observe a significant positive correlation (**p = 0.008) (Fig. 2f).

Mice co-immunized with pADA and 1 μg pBG505-TTT exhibited significantly increased SOSIP-specific IgG2b at D21P2 (*p = 0.0101, mean O.D.=0.933) and a trend of increased SOSIP IgG2b (mean O.D.=1.121) at D14P3 compared to mice immunized with 1 μg pBG505-TTT alone (mean O.D. D21P2 = 0.142, mean O.D. D14P3 = 0.221). Similar to SOSIP-specific IgG and IgG1 responses, SOSIP-specific IgG2b levels plateau at 5 μg pBG505-TTT (mean O.D. D21P2 = 0.360, mean O.D. D14P3 = 0.608) and we observe no further enhancement with pADA (mean O.D. D21P2 = 0.332, mean O.D. D14P3 = 0.900) (Fig. 2c). When correlating SOSIP-specific IgG1 to and IgG2b production, we observe a significant positive correlation (**p = 0.001) between SOSIP-specific IgG1 and IgG2b (Fig. 2e). Regardless of timepoint or immunization, we observed minimal SOSIP-specific IgG3 production with no differences between mice immunized with pBG505-TTT alone or with pADA (Fig. 2d). Collectively, these data demonstrate that pADA enhances the isotype switching of SOSIP-specific Abs and uniquely drives IgG2a expression.

### ADA-1 enhances HIV specific T cell responses

**Figure 3:**
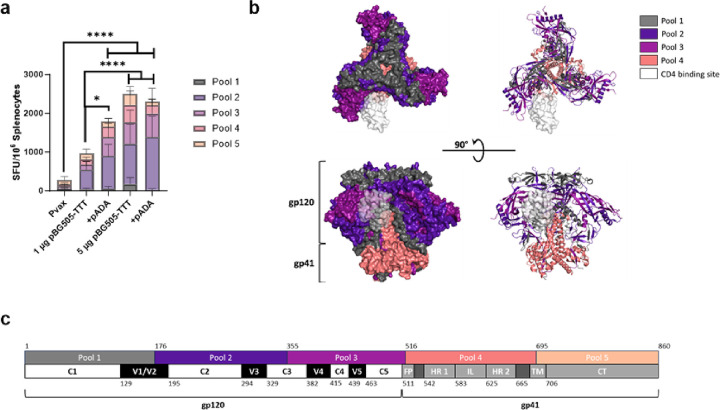
ADA-1 enhances HIV specific T cell responses. Splenocytes from immunized mice (Fig. 1a) were harvested at D14P3 immunizations and stimulated with peptides spanning the HIV BG505 envelope protein. SOSIP specific cellular responses were evaluated via ELISpot assay **(a).** 3D structural **(b)** and linear **(c)** representation of BG505 peptide pools in relation to BG505 protein. Bars represent the mean (n=8 animals per group), error bars represent the standard deviations. *p<0.05, **p<0.01, ***p<0.001, and ****p<0.0001 by two-way ANOVA.

To quantify HIV-BG505 cell mediated immunity, we performed an IFNγ ELISpot assay using splenocytes from immunized mice as outlined in Fig. 1a. Spleens were harvested D14P3 and stimulated with peptide pools encompassing the HIV BG505 protein. HIV specific IFNγ production was quantified by spot forming units (SFUs) per million splenocytes using an ELISpot assay. In line with the humoral responses (Fig. 1–2), mice co-immunized with pADA and 1 μg pBG505-TTT had significantly increased IFNγ SFUs (*p = 0.0455, mean SFU = 1785) compared to mice with 1 μg pBG505-TTT alone (mean SFU = 958). Similar to humoral responses, we observed a plateau in SOSIP-specific IFNγ production in mice immunized with 5 μg pBG505-TTT alone (mean SFU = 2496) or with pADA (mean SFU = 2312) (Fig. 3a). These data suggest that ADA-1 co-delivery can enhance SOSIP-specific cellular IFNγ production and humoral responses (Fig. 1) in a dose sparing manner.

To understand immunogenic epitopes driving T cell responses, we colored the BG505 peptide pools onto the corresponding regions of the BG505 protein using a cryo-EM structure of a BG505 SOSIP.664 trimer (PDB ID: 6V0R). The peptides included in pool 5 have not been structurally resolved on the BG505 protein and are therefore not included. To determine the CD4 binding site, a cryo-EM structure of gp140 bound to the extracellular domains of CD4 (PDB ID: 7T0O) was aligned to the BG505 SOSIP.664 trimer (Fig. 3b–c). T cell responses against pBG505-TTT were primarily driven by the BG505 peptide pool 2 and pool 3. Pool 2 and 3 spanned the majority of the gp120 subunit. Notably, these peptide pools spanned important epitopes of BG505 including variable loops (V1/2, V3, V4 and V5), conserved domains (C2, C3, C4 and C5) and the CD4 binding site (Fig. 3b–c). These responses against peptide pool 2 and pool 3 were not necessarily enhanced with ADA-1, however, these findings demonstrate the ability of pBG505-TTT to drive T cell responses against important epitopes of the BG505 protein. Studies performing T cell epitope mapping of BG505 have been limited but have demonstrated major T cell epitopes within BG505 as C1, C2, C4, C5, V4 in gp120 and HR1 in gp41[42] which is in line with our findings presented here.

### ADA-1 enhances HIV specific T cell polyfunctionality and degranulation

**Figure 4:**
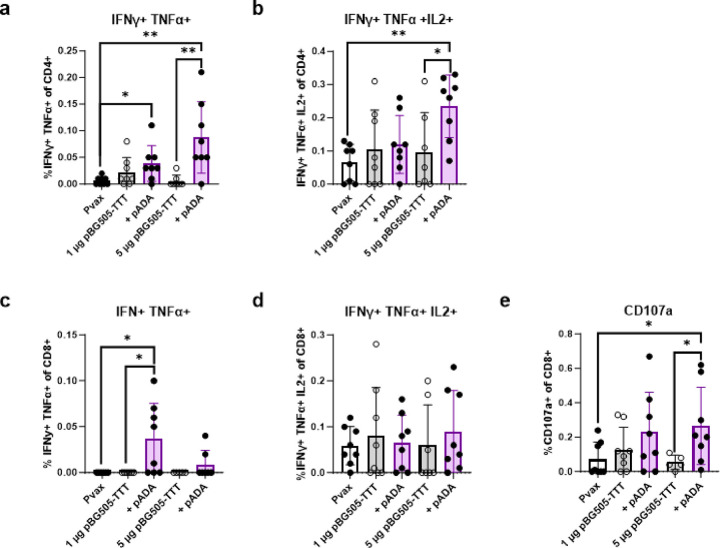
ADA-1 enhances HIV specific T cell polyfunctionality and degranulation. Splenocytes from immunized mice (Fig. 1a) were harvested at D14P3 immunizations and stimulated with peptides spanning the HIV BG505 envelope protein. SOSIP specific cellular responses were evaluated via intracellular cytokine Staining **(a-d).** CD107a expression was evaluated via flow cytometry **(e)**. Data are representative of one experiment. Symbols represent individual animals (n=8 per group). White bars with closed circles represent mice immunized with Pvax. Gray bars with open circles represent animals immunized with bars 1 μg or 5 μg of pBG505-TTT. Purple bars with closed circles represent animals that were immunized with 1 μg or 5 μg of pBG505-TTT + pADA. Bars represent the mean and error bars represent the standard deviations. *p<0.05, **p<0.01, ***p<0.001, and ****p<0.0001 by Mann-Whitney-U test.

To further quantify HIV-BG505 cell-mediated immunity, we performed intracellular cytokine staining (ICS) to determine monofunctionality (IFNγ, TNFα or IL-2 expression, **Supplemental Fig. 3**) and polyfunctionality (IFNγ and TNFα or IFNγ, TNFα and IL-2 expression) of both CD4^+^ (Fig. 4a–b) and CD8^+^ (Fig. 4c–d) T cells using splenocytes from immunized mice as outlined in Fig. 1A. Spleens were harvested D14P3 and stimulated with peptide pools encompassing the HIV BG505 protein. Simultaneously, cells were stained for CD62L and CD44 to quantify central memory (CD62L^+^, CD44^+^) and effector memory (CD62L^−^, CD44^+^) T cells (**Supplemental Fig. 7**). The gating strategy used is shown in **Supplemental Fig. 6**. Cells were also stained for CD107a, a degranulation marker, to evaluate CD8^+^ T cell cytotoxicity (Fig. 4e). The gating strategy used is displayed in **Supplemental Fig. 2**.

When evaluating monofunctionality of CD4^+^ T cells, ADA-1 co-immunization resulted in increased frequencies of CD4^+^ T cells producing IFNγ and TNFα. Mice co-immunized with pADA and 1μg pBG505-TTT has significantly increased frequencies of IFNγ^+^ CD4^+^ T cells compared to mice immunized with 1 μg pBG505-TTT (*p = 0.0351) or Pvax (*p = 0.0256) (**Supplemental Fig. 3a**). Mice co-immunized with pADA and 5 μg pBG505-TTT had significantly increased frequencies of TNFα^+^ CD4^+^ T cells compared to mice immunized with 5 μg pBG505-TTT (*p = 0.0499) or Pvax (**p = 0.0014) (**Supplemental Fig. 3b**). We observed a trend of increased IL2^+^ CD4^+^ T cell frequencies with pADA and 5 μg pBG505-TTT, although not significant when compared to mice receiving 5 μg pBG505-TTT alone (**Supplemental Fig. 3c**).

When evaluating polyfunctionality of CD4^+^ T cells, we found that mice co-immunized with pADA and 5 μg pBG505-TTT had significantly increased frequencies of IFNγ^+^ TNFα^+^ CD4^+^ T cells compared to mice that were immunized with pVax (**p = 0.0047) and 5 μg pBG505-TTT alone (**p = 0.0030) (Fig. 4a). Mice co-immunized with pADA and 1 μg pBG505-TTT had significantly increased frequencies of IFNγ^+^ TNFα^+^ CD4^+^ T cells compared to pVax (*p = 0.0120) and an increasing trend when compared to mice immunized with 1 μg pBG505-TTT alone although not significant (Fig. 4a). Mice co-immunized with pADA and 5 μg pBG505-TTT had significantly increased frequencies of IFNγ^+^ TNFα^+^ IL2^+^ CD4^+^ T cells compared to mice that were immunized with Pvax (**p = 0.0023) 5 μg pBG505-TTT alone (*p = 0.0389) (Fig. 4b).

In the CD8^+^ T cell compartment, we observed no differences in monofunctionality between mice that had been co-immunized with pADA and mice immunized with pBG505 (**Supplemental Fig. 3d-f**). In terms of CD8^+^ T cell polyfunctionality, we observed that mice co-immunized with pADA and 1 μg pBG505-TTT had significantly increased frequencies of IFNγ^+^ TNFα^+^ CD8^+^ T cell compared to mice immunized with Pvax (*p = 0.0430) and 1 μg pBG505-TTT alone (*p = 0.0430) (Fig. 4c). When evaluating CD8^+^ T cell producing IFNγ, TNFα, and IL-2, however, we observed no differences between mice co-immunized with pADA or immunized with pBG505 alone (Fig. 4d). Mice co-immunized with pADA and 5 μg pBG505-TTT had significantly increased frequencies of CD8^+^ T cells expressing the degranulation marker CD107a compared to mice immunized with Pvax (*p = 0.0258) and 5 μg pBG505-TTT alone (*p = 0.0420) (Fig. 4e). Enhanced CD107a expression with pADA co-immunization suggests that ADA-1 increases the ability of CD8^+^ T cell to degranulate and to elicit cytotoxicity [43].

When evaluating central and effector memory T cell responses, we observed no effect on CD4^+^ central memory (CD62L^+^, CD44^+^) T cells (**Supplemental Fig. 7a**) with or without pADA. Mice co-immunized with 1 μg pBG505-TTT (*p = 0.0115) or 5 μg pBG505-TTT (*p = 0.0263) and pADA did have significant increases in frequencies of CD4^+^ effector memory (CD62L^−^, CD44^+^) T cells compared to Pvax immunized mice. These increases with pADA however, were not significant when compared to mice immunized with 1 μg pBG505-TTT or 5 μg pBG505-TTT alone (**Supplemental Fig. 7b**). Similar to CD4^+^ T cell central memory, we observed no effect on CD8^+^ T cell central memory (CD62L^+^, CD44^+^) frequencies (**Supplemental Fig. 7c**) with or without pADA. In contrast to CD8^+^ T cell central memory, we observed a significant increase in frequencies of CD8^+^ effector memory (CD62L^−^, CD44^+^) T cells with pADA co-immunization at both the 1 μg pBG505-TTT (***p = 0.0002) and 5 μg pBG505-TTT (*p = 0.0289) doses compared to mice immunized with 1 μg pBG505-TTT or 5 μg pBG505-TTT alone (**Supplemental Fig. 7d**). Frequencies of CD8^+^ effector memory (CD62L^−^, CD44^+^) T cells were also significantly increased in mice immunized with 1 μg pBG505-TTT and pADA (***p = 0.0002) and 5 μg pBG505-TTT + pADA (**p = 0.0047) doses compared to mice immunized with Pvax.

Taken together, these data demonstrate that ADA-1 enhances HIV specific T cell cytokine production and polyfunctionality of effector T cells. These data also suggest that ADA-1 enhances CD4^+^ and CD8^+^ effector memory T cell responses but has little to no effect on central memory T cell responses.

### ADA-1 enhances HIV specific T cell activation and memory B cell frequencies

To further quantify HIV-BG505 cell mediated immunity, we performed an activation induced marker (AIM) assay and quantified SOSIP-specific memory B cells using flow cytometry. The AIM assay, as previously reported [44], is used to assess antigen specific activation of CD4^+^ T cells through the upregulation of activation markers such as PDL1, OX40 and CD25 upon stimulation. This assay has been described to enrich and monitor antigen specific TFH cells. For these experiments, splenocytes from immunized mice as outlined in Fig. 1a were stimulated with peptide pools encompassing the HIV BG505 protein and PDL1, OX40 and CD25 expression on CD^+^ T cells was quantified via flow cytometry. The gating strategy used is shown in **Supplemental Fig. 4**.

**Figure 5:**
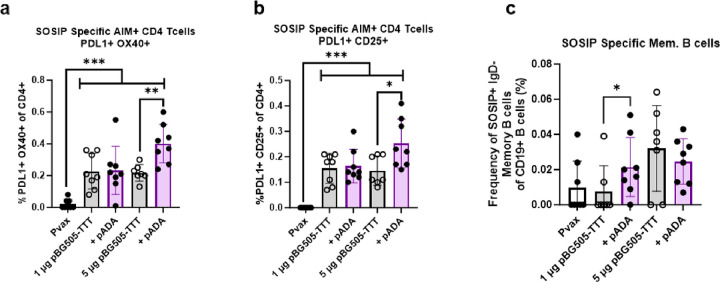
ADA-1 enhances HIV specific T cell activation and memory B cell frequencies. Splenocytes from immunized mice (Fig. 1a) were harvested at D14P3 immunizations and stimulated with peptides spanning the HIV BG505 envelope protein. SOSIP specific cellular responses were evaluated via an activation induced marker (AIM) assay **(a-b)**. Splenocytes from immunized mice (Fig. 1a) were incubated with biotinylated SOSIP protein to evaluate frequencies of SOSIP specific memory B cells **(c)**. Data are representative of one experiment. Symbols represent individual animals (n=8 per group). White bars with closed circles represent mice immunized with Pvax. Gray bars with open circles represent animals immunized with bars 1 μg or 5 μg of pBG505-TTT. Purple bars with closed circles represent animals that were immunized with 1 μg or 5 μg of pBG505-TTT + pADA. Bars represent the mean and error bars represent the standard deviations. *p<0.05, **p<0.01, ***p<0.001, and ****p<0.0001 by Mann-Whitney-U test.

Mice co-immunized with pADA and 5 μg pBG505-TTT had significantly increased frequencies of CD4^+^ T cells expressing PDL1 and OX40 (**p = 0.0022, mean = 0.401%) compared to mice immunized with 5 μg pBG505-TTT alone (mean = 0.217%)(Fig. 5a). Mice co-immunized with pADA and 5 μg pBG505-TTT also had significantly increased frequencies of CD4^+^ T cells expressing PDL1 and CD25 (*p = 0.0280, mean = 0.253%) (Fig. 5b) compared to mice immunized with 5 μg pBG505-TTT alone (mean = 0.146%).

To evaluate SOSIP-specific memory B cell responses, we stained splenocytes from immunized mice as outlined in Fig. 1a with fluorescently labeled multimerized probes specific to SOSIP and defined memory B cells as splenocytes that were CD19^+^IgD^−^ via flow cytometry.

In line with the humoral response (Fig. 1–2), mice co-immunized with pADA and 1 μg pBG505-TTT had significantly increased frequencies of SOSIP-specific memory B cells (*p = 0.0382, mean = 0.022%) compared to mice immunized with 1 μg pBG505-TTT alone (mean = 0.007%). At the 5 μg pBG505-TTT dose, we observed no difference in SOSIP-specific memory B cell frequencies in mice co-immunized with pADA (mean = 0.025%) or immunized with 5 μg pBG505-TTT alone (mean = 0.032%) (Fig. 5c).

Taken together, these data demonstrate that ADA-1 enhances HIV-specific CD4^+^ T activation and SOSIP-specific memory B cells when antigen dose sparing is applied.

### ADA-1 induces qualitative differences in the neutralization capacity of HIV-specific antibodies

**Figure 6:**
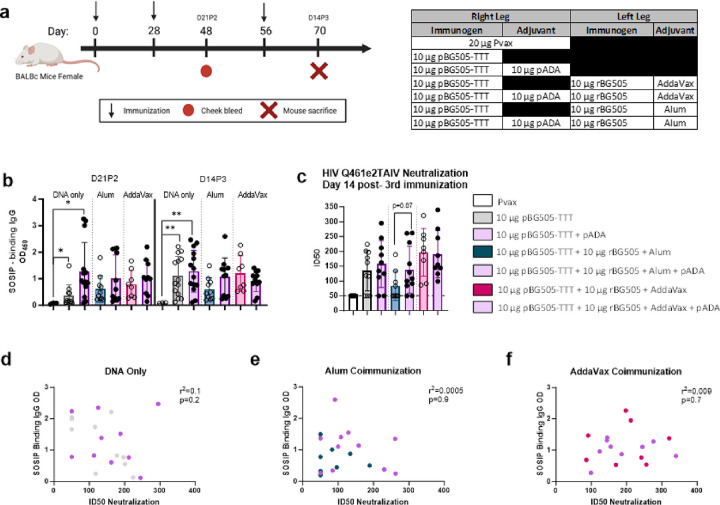
ADA-1 induces qualitative differences in the neutralization capacity of HIV specific Abs. Mice were immunized with 10 μg of pBG505 alone or with 10 μg pADA. A separate group of mice were immunized with both DNA as described and 10 μg of trimeric Env recombinant protein (rBG505) with adjuvants Alum or AddaVax. Mice were bled at D21P2 immunizations prior to sacrifice at D14P3 immunizations **(a)**. Sera was used to determine SOSIP specific IgG via ELISA **(b)** and neutralization **(c).** Pearson correlations were performed for SOSIP specific IgG and neutralization ID50 **(d-f).** Symbols represent individual animals (n=10 per group), bars represent the mean, error bars represent the standard deviations. *p<0.05, **p<0.01, ***p<0.001, and ****p<0.0001 by Mann-Whitney-U test **(b-c)**.

We have previously demonstrated that co-immunization with a plasmid-encoded monomeric gp160 HIV envelope DNA vaccine, recombinant gp160 protein, and pADA in mice resulted in enhanced neutralization of the tier 1A pseudovirus MW965 [12]. In these studies, enhanced neutralization was only the case upon co-immunization with DNA, protein and pADA and was not the case with DNA alone, DNA and protein or DNA and pADA [12]. For this reason, we believed that to see any qualitative difference in Ab responses within a BALB/c mouse model, immunization with DNA, protein and an adjuvant would be necessary. To address this, we immunized mice simultaneously with 10 μg pBG505-TTT alone or with pADA in the right tibialis anterior (TA) muscle and 10 μg rBG505 with alum or with the MF59-like adjuvant AddaVax in the left TA muscle. To understand how pADA performs compared to Alum and AddaVax, we immunized a separate group of mice with 10 μg pBG505-TTT alone or with pADA and no protein co-immunization. As a negative control, we immunized mice with 20μg of Pvax. Mice were immunized thrice and bled on D21P2 and sacrificed at D14P3 (Fig. 6a).

When evaluating SOSIP-specific IgG, across all immunization groups we observed no difference between mice that received pADA or their non-pADA receiving counterparts (Fig. 6b). This is likely due to the higher dose of pBG505-TTT (10 μg) used for this experiment as we observed a plateau in humoral responses with 5 μg pBG505-TTT (Fig. 1–2). We also evaluated SOSIP-specific IgA responses and observed no difference between mice that received pADA or their non-pADA receiving counterparts. We did, however, observe a significant increase in SOSIP-specific IgA in mice immunized with pBG505-TTT and pADA (*p = 0.0307) and mice immunized with pBG505, rBG505, Addavax and pADA (*p = 0.0121) compared to Pvax immunized mice. There was an observed trend toward increased SOSIP-specific IgA in pADA co-immunized mice compared to their non-ADA-1 receiving counterparts although not significant (**Supplemental Fig. 1c**).

When evaluating Ab functionality by pseudoviral neutralization assay, ADA-1 did increase neutralization of the Tier 1A pseudovirus Q461eTAIV to a near significant level (p = 0.07) compared to mice immunized with pBG505-TTT, rBG505, and alum. Mice co-immunized with pBG505-TTT, rBG505, and AddaVax had robust neutralization that was not further improved with pADA (Fig. 6c). We observe no correlation between SOSIP-specific IgG and neutralization ID_50_ demonstrating that the increased Ab quality with pADA is not a direct result of increased Ab magnitude (Fig. 6d–f). We did not observe neutralization in any immunization groups against the Tier 1B virus Q23env17 and the autologous BG505 virus (**Supplementary Table 1–2**) which could in part be due to shortcomings of using wild-type mouse models to evaluate the breadth of antibody neutralization. Collectively these data demonstrate the ability of ADA-1 to enhance the quality of SOSIP-specific Abs by improving neutralization capacity particularly against Tier 1A pseudovirus Q461eTAIV.

## Discussion

We have previously demonstrated that a novel molecular immune modulator, adenosine deaminase-1 (ADA-1) enhances the magnitude and durability of both cellular and humoral vaccine-induced immune responses *in vivo* [12–14]. Additionally, we have shown that ADA-1 is critical to the T follicular helper (TFH) program and that the addition of exogenous ADA-1 enhanced the ability of less efficient pre-TFH to provide help to B cells [20]. We believe ADA-1 is therefore unique as an immune modulator as it targets TFH cells, and TFH cells are central to many aspects critical for effective anti-HIV humoral and cellular responses. TFH cells are known to be critical orchestrators of germinal center reactions which are needed for long-lived humoral and cellular responses. TFH cells are critical to several processes, such as isotype switching, somatic hypermutation, and affinity maturation in B cells, which in turn are needed for the development of bNAbs and therefore critical for an effective anti-HIV humoral response [45]. TFH cells have also been found to boost CD8^+^ T function and enhanced CD8^+^ T function has been implicated in enhanced protection against HIV [33–35]. We therefore hypothesized that ADA-1 combined with pBG505-TTT would result in enhanced magnitude and quality of humoral and cellular HIV specific responses. Our findings demonstrate that ADA-1 has a strong impact on both cellular and humoral immune responses particularly CD8^+^ T cells and qualitative Ab responses which are important contributors to anti-HIV immune responses.

In this study we demonstrated that pADA co-immunization with pBG505-TTT enhances HIV specific humoral and cellular responses. In terms of humoral responses, we observed that pADA co-immunization increases SOSIP-specific Ab magnitude, durability, and isotype switching. This is directly in line and supports our previous findings of pADA enhancing humoral responses to SARS-CoV-2 [13, 14] and monomeric gp160 HIV envelope [12] DNA vaccines. ADA-1 co-immunized mice exhibited a modest increase in ADCD suggesting the induction of nNAbs that are mediating this FcγR-mediated Ab function. ADA-1 could therefore be driving ADCD which has been shown to decrease HIV replication and may play a role in HIV protection [25–30].

ADA-1 induced robust SOSIP-specific IgG, IgG1, IgG2a and IgG2b antibodies at strikingly low doses of pBG505-TTT (1μg) and in a dose-sparing manner. Studies using plasmid encoded MD39, another native-like Env immunogen, required doses of 25μg of DNA to see improved humoral responses [11]. In this study, we found that pADA uniquely induces IgG2a production which was not achieved with pBG505-TTT alone. This is in line with our previous findings of pADA driving TH1-type humoral responses and gene expression profiles [14]. IgG2a production is driven by TH1 responses and, functionally, IgG2a has been correlated with influenza viral clearance and protection against challenge in mice [46] as well as increased overall efficacy of influenza vaccination [47–50], suggesting an important role of IgG2a in viral infections. When considering affinity for Fc receptors to mediate antibody dependent effector functions, mouse IgG2a seems to be a functional analog to human IgG3 [51–53]. HIV infected elite controllers have enhanced antibody functionality including ADCC, monocyte and neutrophil phagocytosis and ADCD primarily coordinated through human IgG3/IgG1 responses [54]. Therefore, the ADA-1 driven enhancement of mouse IgG2a observed could be contributing to enhanced ADCD observed here and suggests ADA-1 could induce human IgG3 with enhanced functionality similar to that observed in elite controllers. Additionally, we found that ADA-1 induced a SOSIP-specific IgA response when co-immunized with pBG505, rBG505, and Addavax suggesting ADA-1 can induce mucosal immunity critical for protection against HIV infection.

This study also demonstrated the ability of ADA-1 to enhance antibody quality. We observed that pADA co-immunization enhanced SOSIP-specific Ab neutralization compared to mice immunized with pBG505, rBG505 and alum. Increased neutralization capacity is presumably a result of increased somatic hypermutation and antibody affinity. This supports our previous findings of increased antibody neutralization and affinity with pADA co-immunization [12–14]. Collectively these findings suggest that ADA-1 is driving key humoral responses that would be necessary for an effective HIV vaccine.

In terms of cellular responses, we demonstrated in this study that ADA-1 enhances HIV-specific CD4^+^ and CD8^+^ T cell responses. ADA-1 co-immunization led to increased HIV-specific IFN-γ production in splenocytes, presumably from both CD4^+^ and CD8^+^ T cells. T cell responses against pBG505-TTT were primarily driven by the BG505 peptide pool 2, which encompassed the variable loop V3 and CD4 binding site. ADA-1 co-immunization also led to increased HIV specific polyfunctionality from both CD4^+^ and CD8^+^ T cells as well as enhanced activation in CD4^+^ T cells and enhanced degranulation (i.e. CD107a expression) in CD8^+^ T cells. We also demonstrated that ADA-1 enhanced frequencies of CD8^+^ effector memory T cells. HIV elite controllers have been found to have CD8^+^ T cells with increased memory and effector potential which importantly provides an increased ability to kill HIV infected cells before progeny virions can be produced [34, 55–57]. Vaccine strategies designed to enhance CD8^+^ T cell frequency and effector function have also demonstrated protective HIV immunity in NHP [35]. Therefore, the increased CD8^+^ effector memory T cells induced by ADA-1 could contribute protective HIV immunity. In terms of B cell responses, we also demonstrate the ability of ADA-1 to enhance SOSIP-specific memory B cell responses. These findings suggest that ADA-1 is driving key cellular responses that would be necessary for an effective HIV vaccine.

A limitation of this study is the shortcoming of using wild-type mouse models to evaluate the breadth of antibody neutralization. Previous studies have shown the difficulty of murine B cells to recognize HIV envelope epitopes necessary for the induction of Tier 2 neutralizing antibodies [8]. While studies in cows have shown BG505 SOSIP immunization to drive broad and potent HIV-specific neutralizing antibody [58], the field has yet to achieve a vaccine that induces bNAb responses in other animal models or in humans [9, 59, 60]. We demonstrated neutralization against the Tier 1A virus Q461e2TAIV; however, we were unable to achieve neutralization in any immunization groups against the Tier 1B virus Q23env17 and or the autologous BG505 virus. Future investigation is needed in other animal models, such as human immunoglobulin, bNAb knock-in mice [61–65], or NHP to evaluate the extent to which ADA-1 can induce bNAb’s for HIV-1.

The current study nonetheless demonstrated an important proof of concept that ADA-1 can act as an immune modulator to enhance HIV-specific humoral and cellular responses in the context of a BG505-SOSIP DNA vaccine. Notably, enhanced humoral and cellular responses with ADA-1 were achieved at very low doses of pBG505-TTT, exemplifying the ability of ADA-1 to be dose-sparing. This study in addition to our previous findings [12–14, 20] demonstrated the ability of ADA-1, when formulated with DNA antigens, to target TFH cells and induce durable, higher-quality antibodies which are critical in the development of protective bNAbs against HIV. Importantly, ADA-1 as an enzyme replacement therapy to treat ADA-SCID has already been approved, is clinically well-tolerated and has a demonstrated safety profile that could facilitate ADA-1 to be fast-tracked for clinical applications [16]. Additionally, BG505-SOSIP is currently being evaluated in several on-going clinical trials (NCT03699241, NCT04177355, NCT05863585, NCT05983874) demonstrating it as a promising vaccine antigen to which responses could be further enhanced with an immune modulator such as ADA-1.

## Data Availability

The datasets used and/or analyzed during the current study are available from the corresponding author upon reasonable request.

## References

[1] Kim J., , Current approaches to HIV vaccine development: a narrative review. Journal of the International AIDS Society, 2021. 24(S7): p. e25793. 34806296 10.1002/jia2.25793 PMC8606871

[2] Sanders R.W., , A next-generation cleaved, soluble HIV-1 Env trimer, BG505 SOSIP.664 gp140, expresses multiple epitopes for broadly neutralizing but not non-neutralizing antibodies. PLoS Pathog, 2013. 9(9): p. e1003618. 24068931 10.1371/journal.ppat.1003618 PMC3777863

[3] Flynn N.M., , Placebo-controlled phase 3 trial of a recombinant glycoprotein 120 vaccine to prevent HIV-1 infection. J Infect Dis, 2005. 191(5): p. 654–65. 15688278 10.1086/428404

[4] Gilbert P.B., , Correlation between immunologic responses to a recombinant glycoprotein 120 vaccine and incidence of HIV-1 infection in a phase 3 HIV-1 preventive vaccine trial. J Infect Dis, 2005. 191(5): p. 666–77. 15688279 10.1086/428405

[5] Pitisuttithum P., , Randomized, double-blind, placebo-controlled efficacy trial of a bivalent recombinant glycoprotein 120 HIV-1 vaccine among injection drug users in Bangkok, Thailand. J Infect Dis, 2006. 194(12): p. 1661–71. 17109337 10.1086/508748

[6] de Taeye S.W., , Immunogenicity of Stabilized HIV-1 Envelope Trimers with Reduced Exposure of Non-neutralizing Epitopes. Cell, 2015. 163(7): p. 1702–15. 26687358 10.1016/j.cell.2015.11.056 PMC4732737

[7] McCoy L.E., , Holes in the Glycan Shield of the Native HIV Envelope Are a Target of Trimer-Elicited Neutralizing Antibodies. Cell Rep, 2016. 16(9): p. 2327–38. 27545891 10.1016/j.celrep.2016.07.074 PMC5007210

[8] Hu J.K., , Murine Antibody Responses to Cleaved Soluble HIV-1 Envelope Trimers Are Highly Restricted in Specificity. Journal of Virology, 2015. 89(20): p. 10383–10398. 26246566 10.1128/JVI.01653-15 PMC4580201

[9] Sanders R.W., , HIV-1 neutralizing antibodies induced by native-like envelope trimers. Science, 2015. 349(6244): p. aac4223. 26089353 10.1126/science.aac4223 PMC4498988

[10] del Moral-Sánchez I., , Triple tandem trimer immunogens for HIV-1 and influenza nucleic acid-based vaccines. 2023. 10.1038/s41541-024-00862-8 PMC10998906 38582771

[11] Xu Z., , Induction of tier-2 neutralizing antibodies in mice with a DNA-encoded HIV envelope native like trimer. Nature Communications, 2022. 13(1): p. 695. 10.1038/s41467-022-28363-z PMC8816947 35121758

[12] Gary E., , Adenosine deaminase-1 enhances germinal center formation and functional antibody responses to HIV-1 Envelope DNA and protein vaccines. Vaccine, 2020. 38(22): p. 3821–3831. 32280045 10.1016/j.vaccine.2020.03.047 PMC7190415

[13] Cusimano G.M., , Improved Durability to SARS-CoV-2 Vaccine Immunity following Coimmunization with Molecular Adjuvant Adenosine Deaminase-1. The Journal of Immunology, 2022: p. ji2200056. 10.4049/jimmunol.2200056 PMC9246991 35750334

[14] Gary E.N., , Adenosine deaminase augments SARS-CoV-2 specific cellular and humoral responses in aged mouse models of immunization and challenge. Frontiers in Immunology, 2023. 14. 10.3389/fimmu.2023.1138609 PMC10043169 36999023

[15] Buckley R.H., Molecular Defects in Human Severe Combined Immunodeficiency and Approaches to Immune Reconstitution. Annual Review of Immunology, 2004. 22(1): p. 625–655. 10.1146/annurev.immunol.22.012703.104614 15032591

[16] Dorsey M.J., , PEGylated Recombinant Adenosine Deaminase Maintains Detoxification and Lymphocyte Counts in Patients with ADA-SCID. J Clin Immunol, 2023. 43(5): p. 951–964. 36840835 10.1007/s10875-022-01426-y PMC10276086

[17] Martinez-Navio J.M., , Immunological dysfunction in HIV-1-infected individuals caused by impairment of adenosine deaminase-induced costimulation of T-cell activation. Immunology, 2009. 128(3): p. 393–404. 20067539 10.1111/j.1365-2567.2009.03121.x PMC2770687

[18] Martinez-Navio J.M., , Adenosine deaminase potentiates the generation of effector, memory, and regulatory CD4+ T cells. J Leukoc Biol, 2011. 89(1): p. 127–36. 20959412 10.1189/jlb.1009696

[19] Pacheco R., , CD26, adenosine deaminase, and adenosine receptors mediate costimulatory signals in the immunological synapse. Proceedings of the National Academy of Sciences of the United States of America, 2005. 102(27): p. 9583–9588. 15983379 10.1073/pnas.0501050102 PMC1172240

[20] Tardif V., , Adenosine deaminase-1 delineates human follicular helper T cell function and is altered with HIV. Nature Communications, 2019. 10(1): p. 823. 10.1038/s41467-019-08801-1 PMC6379489 30778076

[21] Locci M., , Human circulating PD-1+CXCR3-CXCR5+ memory Tfh cells are highly functional and correlate with broadly neutralizing HIV antibody responses. Immunity, 2013. 39(4): p. 758–69. 24035365 10.1016/j.immuni.2013.08.031 PMC3996844

[22] Havenar-Daughton C., , Direct Probing of Germinal Center Responses Reveals Immunological Features and Bottlenecks for Neutralizing Antibody Responses to HIV Env Trimer. Cell Rep, 2016. 17(9): p. 2195–2209. 27880897 10.1016/j.celrep.2016.10.085 PMC5142765

[23] Schoofs T., , HIV-1 therapy with monoclonal antibody 3BNC117 elicits host immune responses against HIV-1. Science, 2016. 352(6288): p. 997–1001. 27199429 10.1126/science.aaf0972 PMC5151174

[24] Pegu A., , Neutralizing antibodies to HIV-1 envelope protect more effectively in vivo than those to the CD4 receptor. Sci Transl Med, 2014. 6(243): p. 243ra88. 10.1126/scitranslmed.3008992 PMC4562469 24990883

[25] Su B., , Update on Fc-Mediated Antibody Functions Against HIV-1 Beyond Neutralization. Front Immunol, 2019. 10: p. 2968. 31921207 10.3389/fimmu.2019.02968 PMC6930241

[26] Parsons M.S., Chung A.W., and Kent S.J., Importance of Fc-mediated functions of anti-HIV-1 broadly neutralizing antibodies. Retrovirology, 2018. 15(1): p. 58. 30134945 10.1186/s12977-018-0438-x PMC6103878

[27] Bournazos S. and Ravetch J.V., Anti-retroviral antibody FcγR-mediated effector functions. Immunol Rev, 2017. 275(1): p. 285–295. 28133801 10.1111/imr.12482 PMC7379163

[28] Mayr L.M., Su B., and Moog C., Non-Neutralizing Antibodies Directed against HIV and Their Functions. Front Immunol, 2017. 8: p. 1590. 29209323 10.3389/fimmu.2017.01590 PMC5701973

[29] Lewis G.K., , Beyond Viral Neutralization. AIDS Res Hum Retroviruses, 2017. 33(8): p. 760–764. 28084796 10.1089/aid.2016.0299 PMC5695748

[30] Holl V., , Nonneutralizing antibodies are able to inhibit human immunodeficiency virus type 1 replication in macrophages and immature dendritic cells. J Virol, 2006. 80(12): p. 6177–81. 16731957 10.1128/JVI.02625-05 PMC1472578

[31] Excler J.L., , Nonneutralizing functional antibodies: a new "old" paradigm for HIV vaccines. Clin Vaccine Immunol, 2014. 21(8): p. 1023–36. 24920599 10.1128/CVI.00230-14 PMC4135913

[32] Laidlaw B.J., , Cooperativity between CD8+ T cells, non-neutralizing antibodies, and alveolar macrophages is important for heterosubtypic influenza virus immunity. PLoS Pathog, 2013. 9(3): p. e1003207. 23516357 10.1371/journal.ppat.1003207 PMC3597515

[33] Zander R., , Tfh-cell-derived interleukin 21 sustains effector CD8+ T cell responses during chronic viral infection. Immunity, 2022. 55(3): p. 475–493.e5. 35216666 10.1016/j.immuni.2022.01.018 PMC8916994

[34] Monel B., , HIV Controllers Exhibit Effective CD8(+) T Cell Recognition of HIV-1-Infected Non-activated CD4(+) T Cells. Cell Rep, 2019. 27(1): p. 142–153.e4. 30943397 10.1016/j.celrep.2019.03.016 PMC6449512

[35] Picker L.J., , Programming cytomegalovirus as an HIV vaccine. Trends Immunol, 2023. 44(4): p. 287–304. 36894436 10.1016/j.it.2023.02.001 PMC10089689

[36] Steichen J.M., , HIV Vaccine Design to Target Germline Precursors of Glycan-Dependent Broadly Neutralizing Antibodies. Immunity, 2016. 45(3): p. 483–496. 27617678 10.1016/j.immuni.2016.08.016 PMC5040827

[37] Torrents de la Peña, A., , Improving the Immunogenicity of Native-like HIV-1 Envelope Trimers by Hyperstabilization. Cell Rep, 2017. 20(8): p. 1805–1817. 28834745 10.1016/j.celrep.2017.07.077 PMC5590011

[38] Smith T.R.F., , Immunogenicity of a DNA vaccine candidate for COVID-19. Nature Communications, 2020. 11(1): p. 2601. 10.1038/s41467-020-16505-0 PMC7239918 32433465

[39] del Moral-Sánchez I., , High thermostability improves neutralizing antibody responses induced by native-like HIV-1 envelope trimers. npj Vaccines, 2022. 7(1): p. 27. 35228534 10.1038/s41541-022-00446-4 PMC8885667

[40] Malherbe D.C., , Sequential Immunization with a Subtype B HIV-1 Envelope Quasispecies Partially Mimics the In Vivo Development of Neutralizing Antibodies. Journal of Virology, 2011. 85(11): p. 5262–5274. 21430056 10.1128/JVI.02419-10 PMC3094990

[41] Fischinger S., , A high-throughput, bead-based, antigen-specific assay to assess the ability of antibodies to induce complement activation. Journal of Immunological Methods, 2019. 473: p. 112630. 31301278 10.1016/j.jim.2019.07.002 PMC6722412

[42] Schiffner T., , Structural and immunologic correlates of chemically stabilized HIV-1 envelope glycoproteins. PLOS Pathogens, 2018. 14(5): p. e1006986. 29746590 10.1371/journal.ppat.1006986 PMC5944921

[43] Aktas E., , Relationship between CD107a expression and cytotoxic activity. Cell Immunol, 2009. 254(2): p. 149–54. 18835598 10.1016/j.cellimm.2008.08.007

[44] Reiss S., , Comparative analysis of activation induced marker (AIM) assays for sensitive identification of antigen-specific CD4 T cells. PloS one, 2017. 12(10): p. e0186998. 29065175 10.1371/journal.pone.0186998 PMC5655442

[45] Bettini E. and Locci M., SARS-CoV-2 mRNA Vaccines: Immunological Mechanism and Beyond. Vaccines, 2021. 9(2). 10.3390/vaccines9020147 PMC7918810 33673048

[46] Huber V.C., , Distinct Contributions of Vaccine-Induced Immunoglobulin G1 (IgG1) and IgG2a Antibodies to Protective Immunity against Influenza. Clinical and Vaccine Immunology, 2006. 13(9): p. 981–990. 16960108 10.1128/CVI.00156-06 PMC1563571

[47] Hovden A.O., Cox R., and Haaheim L., Whole influenza virus vaccine is more immunogenic than split influenza virus vaccine and induces primarily an IgG2a response in BALB/c mice. Scandinavian journal of immunology, 2005. 62(1): p. 36–44. 10.1111/j.1365-3083.2005.01633.x 16092921

[48] Huber V.C., , Fc receptor-mediated phagocytosis makes a significant contribution to clearance of influenza virus infections. The Journal of Immunology, 2001. 166(12): p. 7381–7388. 11390489 10.4049/jimmunol.166.12.7381

[49] Moran T.M., , Th2 responses to inactivated influenza virus can be converted to Th1 responses and facilitate recovery from heterosubtypic virus infection. The Journal of infectious diseases, 1999. 180(3): p. 579–585. 10438342 10.1086/314952

[50] Arulanandam B.P., O’Toole M., and Metzger D.W., Intranasal interleukin-12 is a powerful adjuvant for protective mucosal immunity. The Journal of infectious diseases, 1999. 180(4): p. 940–949. 10479116 10.1086/314996

[51] Bruhns P., Properties of mouse and human IgG receptors and their contribution to disease models. Blood, 2012. 119(24): p. 5640–5649. 22535666 10.1182/blood-2012-01-380121

[52] Nimmerjahn F., , FcgammaRIV: a novel FcR with distinct IgG subclass specificity. Immunity, 2005. 23(1): p. 41–51. 16039578 10.1016/j.immuni.2005.05.010

[53] Heß R., , Glycosylation of HIV Env Impacts IgG Subtype Responses to Vaccination. Viruses, 2019. 11(2). 10.3390/v11020153 PMC6410111 30781796

[54] Ackerman M.E., , Polyfunctional HIV-Specific Antibody Responses Are Associated with Spontaneous HIV Control. PLOS Pathogens, 2016. 12(1): p. e1005315. 26745376 10.1371/journal.ppat.1005315 PMC4706315

[55] Collins D.R., Gaiha G.D., and Walker B.D., CD8+ T cells in HIV control, cure and prevention. Nature Reviews Immunology, 2020. 20(8): p. 471–482. 10.1038/s41577-020-0274-9 PMC7222980 32051540

[56] Yang O.O., , Efficient lysis of human immunodeficiency virus type 1-infected cells by cytotoxic T lymphocytes. Journal of virology, 1996. 70(9): p. 5799–5806. 8709196 10.1128/jvi.70.9.5799-5806.1996 PMC190594

[57] Sacha J.B., , Gag-specific CD8+ T lymphocytes recognize infected cells before AIDS-virus integration and viral protein expression. The Journal of Immunology, 2007. 178(5): p. 2746–2754. 17312117 10.4049/jimmunol.178.5.2746 PMC4520734

[58] Heydarchi B., , Broad and ultra-potent cross-clade neutralization of HIV-1 by a vaccine-induced CD4 binding site bovine antibody. Cell Rep Med, 2022. 3(5): p. 100635. 35584627 10.1016/j.xcrm.2022.100635 PMC9133467

[59] Pauthner M., , Elicitation of Robust Tier 2 Neutralizing Antibody Responses in Nonhuman Primates by HIV Envelope Trimer Immunization Using Optimized Approaches. Immunity, 2017. 46(6): p. 1073–1088.e6. 28636956 10.1016/j.immuni.2017.05.007 PMC5483234

[60] Ng’uni T., Chasara C., and Ndhlovu Z.M., Major Scientific Hurdles in HIV Vaccine Development: Historical Perspective and Future Directions. Frontiers in Immunology, 2020. 11. 10.3389/fimmu.2020.590780 PMC7655734 33193428

[61] Verkoczy L., Humanized Immunoglobulin Mice: Models for HIV Vaccine Testing and Studying the Broadly Neutralizing Antibody Problem. Adv Immunol, 2017. 134: p. 235–352. 28413022 10.1016/bs.ai.2017.01.004 PMC5914178

[62] Dosenovic P., , Immunization for HIV-1 Broadly Neutralizing Antibodies in Human Ig Knockin Mice. Cell, 2015. 161(7): p. 1505–15. 26091035 10.1016/j.cell.2015.06.003 PMC4604566

[63] Briney B., , Tailored Immunogens Direct Affinity Maturation toward HIV Neutralizing Antibodies. Cell, 2016. 166(6): p. 1459–1470.e11. 27610570 10.1016/j.cell.2016.08.005 PMC5018249

[64] Escolano A., , Sequential Immunization Elicits Broadly Neutralizing Anti-HIV-1 Antibodies in Ig Knockin Mice. Cell, 2016. 166(6): p. 1445–1458.e12. 27610569 10.1016/j.cell.2016.07.030 PMC5019122

[65] Tian M., , Induction of HIV Neutralizing Antibody Lineages in Mice with Diverse Precursor Repertoires. Cell, 2016. 166(6): p. 1471–1484.e18. 27610571 10.1016/j.cell.2016.07.029 PMC5103708

